# Efficacy and Safety of Integrated Traditional Chinese Medicine and Western Medicine on the Treatment of Rheumatoid Arthritis: A Meta-Analysis

**DOI:** 10.1155/2020/4348709

**Published:** 2020-04-02

**Authors:** Qi Xing, Ling Fu, Zhichao Yu, Xueping Zhou

**Affiliations:** The First Clinical Medical College, Nanjing University of Chinese Medicine, Nanjing 210023, China

## Abstract

**Objective:**

Integrated therapy of traditional Chinese medicine (TCM) and Western medicine (WM) has gradually been applied to the treatment of rheumatoid arthritis (RA). Recently published studies have provided a wealth of data and information about the effectiveness of combination treatments, but high-quality evidence-based meta-analysis on this issue is not available yet. This study was conducted to compare and evaluate the efficacy and safety of the integrated therapy for RA.

**Methods:**

PubMed, EMBASE, and the Cochrane Library were searched up to January 2020. Randomized controlled trials (RCTs) that compared the efficacy and safety of integrative TCM-WM with WM alone for RA were included. The outcome measures contained therapeutic effects (TEs), tender joint count (TJC), swollen joint count (SJC), duration of morning stiffness (DMS), grip strength (GS), disease activity score in 28 joints (DAS28), rheumatoid factor (RF), anti-cyclic peptide containing citrulline (anti-CCP), erythrocyte sedimentation rate (ESR), C-reactive protein (CRP), and adverse events (AEs) to assess the efficacy and safety of different treatments.

**Results:**

A total of 20 RCTs with 2269 patients met the inclusion criteria. TCM used in these studies included Chinese herbal decoctions and tablets or capsules made from herbs and their extracts, while WM included disease-modifying antirheumatic drugs (DMARDs), nonsteroidal anti-inflammatory drugs (NSAIDs), and glucocorticoids (GC). Compared with patients receiving WM treatment alone, patients with integrative TCM-WM treatment showed better TEs (OR = 3.03, 95% CI [2.36, 3.88]). The integrative treatment group showed reductions in TJC (MD = −1.17, 95% CI [−2.12, −0.21]), SJC (MD = −0.87, 95% CI [−1.85, 0.10]), DMS (SMD = −0.69, 95% CI [−0.98, −0.41]), DAS28 (MD = −0.43, 95% CI [−0.57, −0.29]), RF (SMD = −0.59, 95% CI [−0.91, −0.27]), anti-CCP (SMD = −0.21, 95% CI [−0.36, −0.06]), ESR (MD = −8.36, 95% CI [−12.60, −4.12]), and CRP (MD = −6.73, 95% CI [−9.38, −4.08]), and increment in GS (SMD = 0.12, 95% CI [−0.63, 0.87]). AEs, especially gastrointestinal disorders, abnormal liver function, leukopenia, skin allergies and rashes, headaches and dizziness, and alopecia, significantly decreased (OR = 0.37, 95% CI [0.29, 0.47]) in the integrative treatment group.

**Conclusions:**

The findings of this meta-analysis indicate that integrative TCM-WM could obtain effective and safe results in the treatment of RA. Using TCM as an adjunctive therapy in RA has great prospects for further development.

## 1. Introduction

Rheumatoid arthritis (RA) is one of the most prevalent chronic systemic autoimmune diseases [1]. It is characterized by synovial membrane inflammation and hyperplasia, autoantibody production, cartilage and bone destruction, and systemic features [2]. The typical symptoms of RA are pain and swelling in the joints of hands and feet, accompanied by morning stiffness of the affected joints; large joints including shoulder, elbow, knee, and ankle joints could also be injured [3]. RA has a relatively constant incidence of 0.5% to 1% [4], and population-based epidemiologic studies consistently reveal that family history of RA increases the risk of the onset of it by 3–5 times [5].

The therapeutic targets of RA are focusing on reducing joint inflammation and pain, maximizing joint function, and preventing from articular destruction and deformity. Treatment regimens are composed of medications, weight-bearing exercise, health education, and rest [6]. Western medicine (WM) treatment for RA mainly includes nonsteroidal anti-inflammatory drugs (NSAIDs), disease-modifying antirheumatic drugs (DMARDs), glucocorticoids (GC), and biological agents. NSAIDs are not used to control the disease progression of RA [7]. Methotrexate (MTX) among DMARDs is still the first-line choice for treating RA recommended by the international guidelines [8], but researches indicated generally low remission rates with MTX monotherapy [9]. GC is the most potent disease-modifying drug in clinic at present, but its chronic use could cause osteoporosis, osteonecrosis, and other hazards [10]. Biological agents are expensive and their long-term effects are still controversial, though they have a positive effect on symptom reduction of RA [11, 12].

China has abundant botanical resources which have been widely used in RA treatment [13–15]. *Tripterygium wilfordii* Hook. f., *Aconitum carmichaelii* Debx., and *Curcuma longa* L. represent a few of the many medicines of botanical origin for RA in traditional Chinese medicine (TCM), which may have a positive effect not only on the symptoms but also on the disease progression [16–18]. Formula is the main category of herbal remedies. Guizhi-Shaoyao-Zhimu Decoction is a representative prescribed formula to treat RA. A synthetic approach [19] that combined drug target prediction, network analysis, and experimental validation indicated that Guizhi-Shaoyao-Zhimu Decoction may partially attenuate RA by means of reversing inflammation-immune system disequilibrium and regulating the HDAC1-HSP90AA1-NFKB2-IKBKB-TNF-*α* signaling axis. As one of novel Chinese patent medicines, Xinfeng capsule shows benefits in alleviating joint pain, swelling, and early morning stiffness, and it could also ameliorate extra-articular manifestations such as anemia, platelet disorder, lipid metabolism disturbance, abnormal cardiopulmonary function, depression, and quality of life with few adverse reaction [20, 21]. Many effective ingredients of antirheumatic Chinese herbs have been found to inhibit RA development and some of the effective extracts have been verified. Luo et al. [22] summarized evidences on the efficacy and safety of clinical application of tripterygium glycosides and total glucosides of paeony, suggesting that they might be potential beneficial complementary and alternative medicines for RA patients. *Artemisia asiatica* has a long history of ethnopharmacological use in Asian countries such as China, Korea, and Japan, and a novel antioxidative and anti-inflammatory formulation prepared from the ethanol extracts of *Artemisia asiatica* named DA-9601 is now on sale in South Korea [23, 24]. A recent study [25] has shown that DA-9601 injection reduced arthritis scores in collagen-induced arthritis mice; moreover, eupatilin, the main active component of DA-9601, could markedly downregulate the expression of inflammatory cytokines and suppress the differentiation of osteoclasts, indicating that DA-9601 and eupatilin are candidate anti-inflammatory agents.

TCM has special superiorities in reducing the adverse reactions of WM and improving its curative effect [26, 27]. So, the combination of TCM and conventional WM provides a new approach for the improvement of quality of life and disease control of RA patients. Many studies showed that the integrated TCM-WM therapy has a positive effect on the treatment of RA. However, due to the small sizes of multisamples and uneven quality of articles, it is difficult to draw reliable conclusions based on small-sample randomized controlled trials (RCTs). Therefore, we conducted this meta-analysis aiming to systematically evaluate the efficacy and safety of integrated TCM-WM versus WM monotherapy for the treatment of RA. We supposed that this research could provide the evidence for the superiority of treating RA with integrative medicine.

## 2. Methods

### 2.1. Search Strategy

Associated studies from inception to January 2020 were retrieved in the following electronic databases: PubMed, EMBASE, and the Cochrane Library. The search strategies for each database are presented in the Supplementary [Supplementary-material supplementary-material-1]. In addition, the reference lists of relevant publications were manually searched to find additional studies. The searches were independently performed by two authors.

### 2.2. Inclusion and Exclusion Criteria

The following were included: (1) studies published in either English or Chinese language; (2) participating patients diagnosed with RA in accordance with the 1987 American Rheumatism Association (1987 ARA) or the 2010 American College of Rheumatology and European Union League Against Rheumatism (2010 ACR/EULAR) diagnostic criteria; (3) experimental groups (EGs) treated with a combination of TCM and WM, while control groups (CGs) treated only with WM; (4) RCTs; and (5) detailed data of at least 1 relevant outcome.

The following were excluded: (1) participants not diagnosed with RA according to the diagnostic criteria mentioned above; (2) participants restricted to special crowd (e.g., the elderly and juveniles); (3) EGs treated only with TCM; (4) duplicative data; (5) incomplete or unavailable data; and (6) reviews, conference abstracts, and case reports.

### 2.3. Types of Outcome Measures

The primary outcomes analyzed in this meta-analysis were therapeutic effects (TEs) and adverse events (AEs). The secondary outcomes were tender joint count (TJC), swollen joint count (SJC), duration of morning stiffness (DMS), grip strength (GS), disease activity score in 28 joints (DAS28), rheumatoid factor (RF), anti-cyclic peptide containing citrulline (anti-CCP), erythrocyte sedimentation rate (ESR), and C-reactive protein (CRP).

TEs were associated with the improvements of clinical symptoms and laboratory indexes, and the most used remission criterion was ACR20/50/70 [28]. ACR20 signified 20% improvements in TJC and SJC as well as 20% improvements in at least 3 of the 5 following items: (1) patient assessment of pain; (2) patient global assessment of disease activity; (3) physician global assessment of disease activity; (4) health assessment questionnaire (HAQ); and (5) acute-phase reactants (ESR and CRP). ACR50 and ACR70 represented 50% and 70% improvements, respectively. The response to treatments was evaluated excellent if the overall improvement of ACR70 was 70%; good if the overall improvement of ACR50 was between 50% and 69%; moderate if the overall improvement of ACR20 was between 20% and 49%; and poor if the treatment did not meet the ACR20 standard. TEs were calculated from the number of excellent, good, and moderate results.

All data were acquired directly from the original studies. Dichotomous variables (TEs and AEs) were expressed as absolute numbers, and continuous data (TJC, SJC, DMS, GS, DAS28, RF, anti-CCP, ESR, and CRP) were expressed as mean with standard deviation for further analysis.

### 2.4. Data Extraction and Quality Assessment

The relevant data were selected and extracted independently by two authors, including names of authors, publication years, sample sizes, ages, genders, courses of the disease, intervention methods, durations of intervention, and outcome indexes. Disagreements were resolved by discussing with a third investigator.

The qualities of the studies included were evaluated by each author on the basis of the Cochrane collaboration's tool [29] for bias risk assessing. The assessments were performed on the following: (1) random sampling method; (2) allocation concealment method; (3) blinding of subjects and experimenters; (4) blinding of outcome assessment; (5) the completion of outcome data; (6) report selection; and (7) other bias, such as specific research designs that could affect the overall outcomes. The results of the 7 items above were assessed as low risk, unclear, or high risk.

### 2.5. Statistical Analysis

All included studies were analyzed with Review Manager 5.3 software (The Cochrane Collaboration, Copenhagen, Denmark). Odds ratios (OR) and 95% confidence intervals (CI) were calculated for dichotomous data, while mean differences (MD), standardized mean differences (SMD), and 95% CI were calculated for continuous data. Heterogeneity was statistically assessed using the chi-squared test and the *I*^2^ statistic, and *I*^2^ > 50% indicated obvious heterogeneity among trials [30]. The analysis was carried out by the use of a random-effect model if *P* < 0.1 or *I*^2^ > 50% but a fixed-effect model if *P* ≥ 0.1 or *I*^2^ ≤ 50%. Descriptive approaches would be adopted if the data were insufficient. Publication bias was detected using funnel plot.

## 3. Results

### 3.1. Study Search and Selection

**Figure 1:**
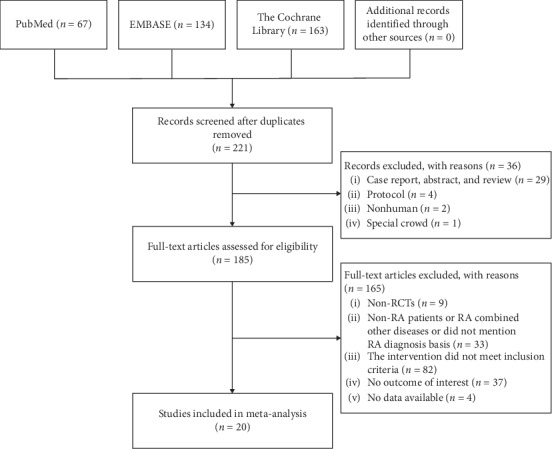
Flow diagram of study selection process.

Initially 364 publications were identified, including 67 articles from PubMed, 134 articles from EMBASE, 163 articles from the Cochrane Library, and no record from manual search. After exclusion of duplicates, 221 studies were screened. Through further evaluation, 20 studies (Wu et al. [31]; Lu et al. [32]; Zhao and Liu [33]; Liu et al. [34]; Li et al. [35]; Lin et al. [36]; Zhao and Wang [37]; Huang et al. [38]; Yu and Yu [39]; Wang et al. [40]; Chen et al. [41]; Wang and Tao [42]; Qian et al. [43]; Jiang et al. [44]; Zhang et al. [45]; Wang [46]; Chen [47]; Du et al. [48]; Yang et al. [49]; and Huang et al. [50]) met the predefined inclusion criteria and were included in this meta-analysis. The general procedure for study selection is summarized in Figure 1.

### 3.2. Description of the Studies

Table 1 summarized the characteristics of the 20 included trials which were studies. There were a total of 2269 cases over all included studies, 2214 (1192 cases for oral TCM combined with WM and 1022 cases for oral WM alone) of them completed the studies. The studies were published between 2001 and 2019, and they were all carried out in China. Sixteen studies were published in Chinese, while 4 studies were in English. Fifteen studies [31–43, 47, 48] used the 1987 ARA diagnostic criteria, including 2 studies [38, 48] combined with the 2010 ACR/EULAR criteria, while 5 studies [44–46, 49, 50] used the 2010 ACR/EULAR criteria. All of the RCTs demonstrated no significant difference in baseline characteristics between experimental and control groups. Of these RCTs, the study population of Huang et al. [38] comprised 28 male patients and 52 female patients with mean age of 36.8 ± 9.3 years and mean disease course of 3.7 ± 2.3 years; the study of Chen et al. [41] comprised 31 male and 165 female participants with mean age of 44.6 ± 13.3 years, including those who had severe adverse reactions and withdrew their consents. The interventions were limited to Chinese herbal medicine and the conventional WM. TCM used in these studies included Chinese herbal decoctions and tablets or capsules made from herbs such as Qingbi Tablet, Kunxian Capsule, and Xinfeng Capsule, or their extracts such as tripterygium glycosides, total glucosides of paeony, sinomenine and the extract of *Artemisia annua* L. WM included DMARDs, NSAIDs, and GC, and the most common of these was MTX. The groups treated with WM only were considered to be the control groups. The foremost outcomes of the included studies were TEs, and all of these studies described them. Eleven studies mentioned the TJC with 10 [31, 32, 34, 36, 38–40, 42, 49, 50] conforming to the desired form of data; 11 mentioned the SJC with 10 [31, 32, 34, 36,38–40, 42, 49, 50] meeting requirements; 9 mentioned the DMS with 9 [31–33, 36, 38–40, 42, 50] meeting requirements; 6 mentioned the GS with 5 [31–33, 39, 40] meeting requirements; 8 mentioned the DAS28 with 5 [38, 40, 44, 48, 50] meeting requirements; 13 reported the effects on RF with 12 [31–33, 36–39, 42, 44, 45, 48, 49] meeting requirements; 8 reported the effects on anti-CCP with 7 [33, 36, 37, 44, 45, 48, 49] meeting requirements; 17 studies reported the effects on ESR with 15 [31–34, 36–40, 42, 44, 45, 48–50] meeting requirements; and 15 reported the effects on CRP with 14 [32–34, 36–40, 42, 44, 45, 48–50] meeting requirements. In addition, 18 [31–38,40–46, 48–50] of these studies discussed the AEs in detail.

### 3.3. Risk of Bias Assessment

**Figure 2:**
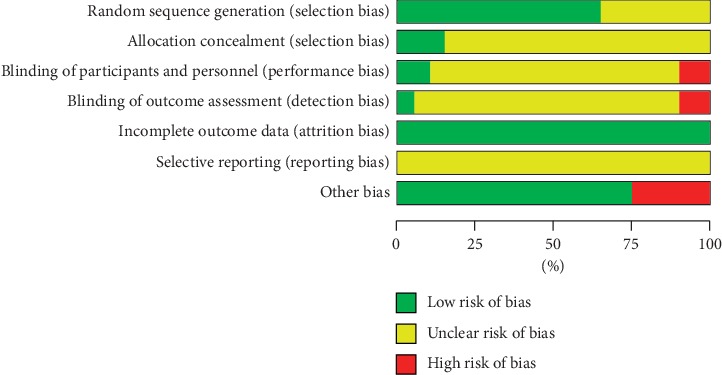
Risk of bias graph.

**Figure 3:**
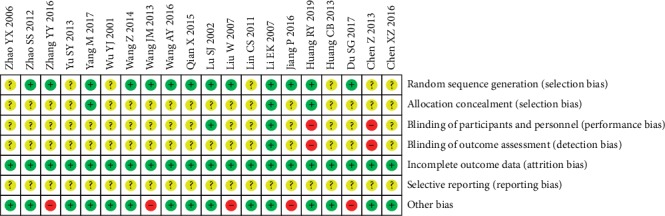
Risk of bias summary.

A summary of the risks of bias in the 20 studies included in the meta-analysis is presented in Figures 2 and 3. For most of the items in the included trials, the risks of bias were low or unclear. All the studies included were described as RCTs; among them, 13 studies [32, 34, 35, 37, 40, 42–46, 48–50] adequately represented the random methods. Allocation concealment and blinding methods were poorly reported. Only 2 trials [35, 50] mentioned allocation concealment methods; others did not specify whether allocation concealment was performed, so the risks of bias in allocation concealment of them were unknown. Two trials [41, 50] were open-label with high risks in performance and detection biases. Regarding incomplete data, which is attrition bias, the authors judged that there was no missing data or that the reasons for the missing outcome indicators could not possibly be related to the true value of the outcomes. Since original study protocols and adequate relevant information were not available to assess selective reporting, all trials were considered to have unclear risks in reporting bias. Five trials [34, 40, 44, 45, 48] were judged at high risk in other bias, for they only focused on specific syndrome types or disease stages of RA, while the others were at low risk.

### 3.4. Effects of Interventions

#### 3.4.1. Clinical Therapeutic Efficacy

**Figure 4:**
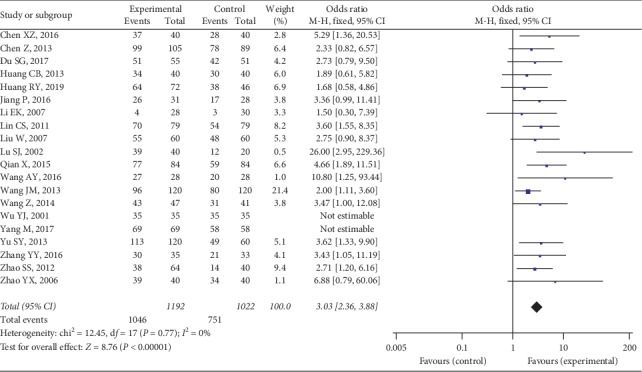
TEs between two groups.

All of the studies demonstrated TEs of the integrated TCM-WM compared with WM only for RA. There was no significant heterogeneity (*I*^2^ = 0%, *P* = 0.77). Therefore, the analysis used a fixed-effect model. The outcome indicated that TEs in the experimental group were significantly better than in the control group (OR = 3.03, 95% CI [2.36, 3.88], *P* < 0.00001) (Figure 4).

#### 3.4.2. Clinical Symptoms

**Figure 5:**
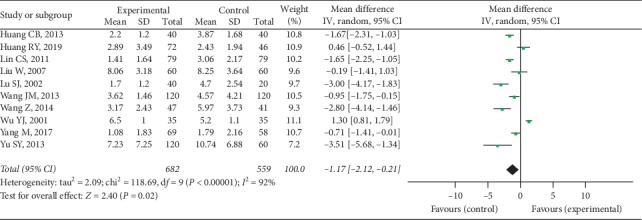
TJC between two groups.

Ten trials provided available TJC data with 682 cases in the experimental group and 559 cases in the control group, and a random-effect model was conducted to analyze the data (*I*^2^ = 92%, *P* < 0.00001). A significant difference was discovered in TJC between 2 groups (MD = −1.17, 95% CI [−2.12, −0.21], *P* = 0.02), as shown in Figure 5.

**Figure 6:**
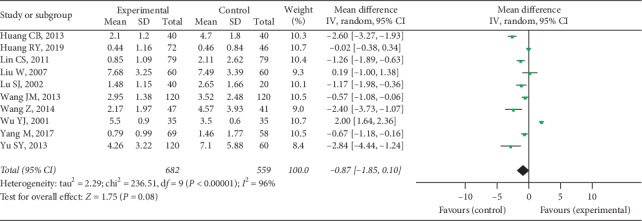
SJC between two groups.

Ten trials provided available SJC data with 682 cases in the experimental group and 559 cases in the control group, and a random-effect model was conducted to analyze the data (*I*^2^ = 96%, *P* < 0.00001). A significant difference was discovered in SJC between 2 groups (MD = −0.87, 95% CI [−1.85, 0.10], *P* = 0.08), as shown in Figure 6.

**Figure 7:**
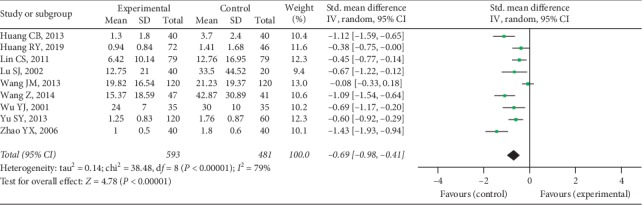
DMS between two groups.

Nine trials provided available DMS data with 593 cases in the experimental group and 481 cases in the control group, and a random-effect model was conducted to analyze the data (*I*^2^ = 79%, *P* < 0.00001). A significant difference was discovered in DMS between 2 groups (SMD = -0.69, 95% CI [−0.98, −0.41], *P* < 0.00001), as shown in Figure 7.

**Figure 8:**
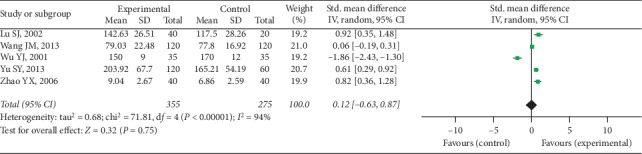
GS between two groups.

Five trials provided available GS data with 355 cases in the experimental group and 275 cases in the control group, and a random-effect model was conducted to analyze the data (*I*^2^ = 94%, *P* < 0.00001). A significant difference was discovered in GS between 2 groups (SMD = 0.12, 95% CI [−0.63, 0.87], *P*=0.75), as shown in Figure 8.

**Figure 9:**
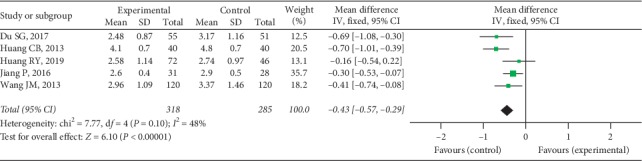
DAS28 between two groups.

Five trials provided available DAS28 data with 318 cases in the experimental group and 285 cases in the control group, and a fixed-effect model was conducted to analyze the data (*I*^2^ = 48%, *P*=0.10). A significant difference was discovered in DAS28 between 2 groups (MD = −0.43, 95% CI [−0.57, −0.29], *P* < 0.00001), as shown in Figure 9.

#### 3.4.3. Laboratory Indexes

**Figure 10:**
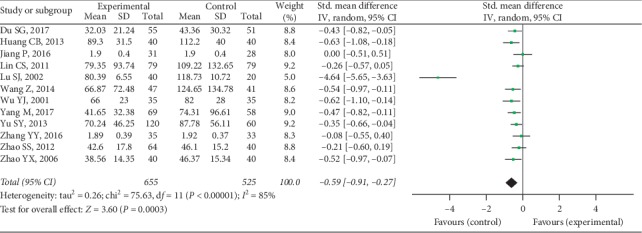
RF between two groups.

Twelve trials provided available RF data with 655 cases in the experimental group and 525 cases in the control group, and a random-effect model was conducted to analyze the data (*I*^2^ = 85%, *P* < 0.00001). A significant difference was discovered in RF between 2 groups (SMD = −0.59, 95% CI [−0.91, −0.27], *P* = 0.0003), as shown in Figure 10.

**Figure 11:**
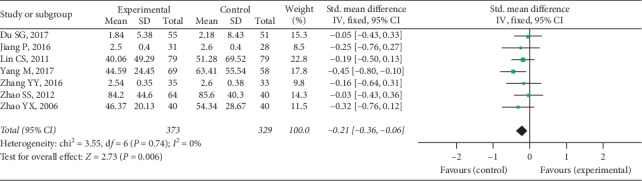
Anti-CCP between two groups.

Seven trials provided available anti-CCP data with 373 cases in the experimental group and 329 cases in the control group, and a fixed-effect model was conducted to analyze the data (*I*^2^ = 0%, *P*=0.74). A significant difference was discovered in anti-CCP between 2 groups (SMD = −0.21, 95% CI [−0.36, −0.06], *P*=0.006), as shown in Figure 11.

**Figure 12:**
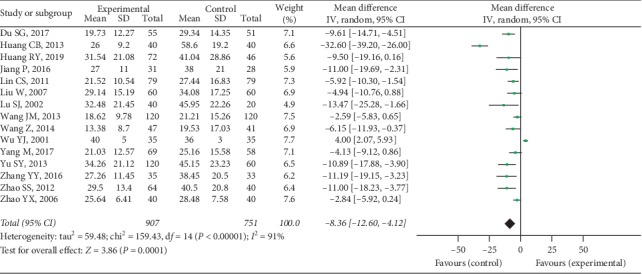
ESR between two groups.

Fifteen trials provided available ESR data with 907 cases in the experimental group and 751 cases in the control group, and a random-effect model was conducted to analyze the data (*I*^2^ = 91%, *P* < 0.00001). A significant difference was discovered in ESR between 2 groups (MD = −8.36, 95% CI [−12.60, −4.12], *P* = 0.0001), as shown in Figure 12.

**Figure 13:**
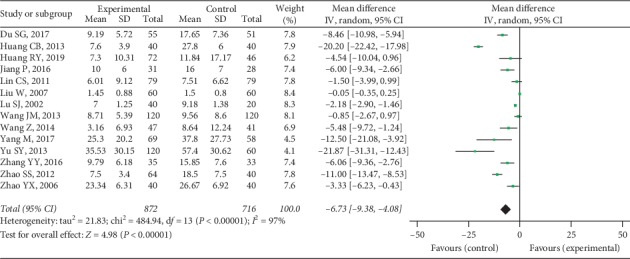
CRP between two groups.

Fourteen trials provided available CRP data with 872 cases in the experimental group and 716 cases in the control group, and a random-effect model was conducted to analyze the data (*I*^2^ = 97%, *P* < 0.00001). A significant difference was discovered in CRP between 2 groups (MD = −6.73, 95% CI [−9.38, −4.08], *P* < 0.00001), as shown in Figure 13.

#### 3.4.4. Adverse Drug Reactions

**Figure 14:**
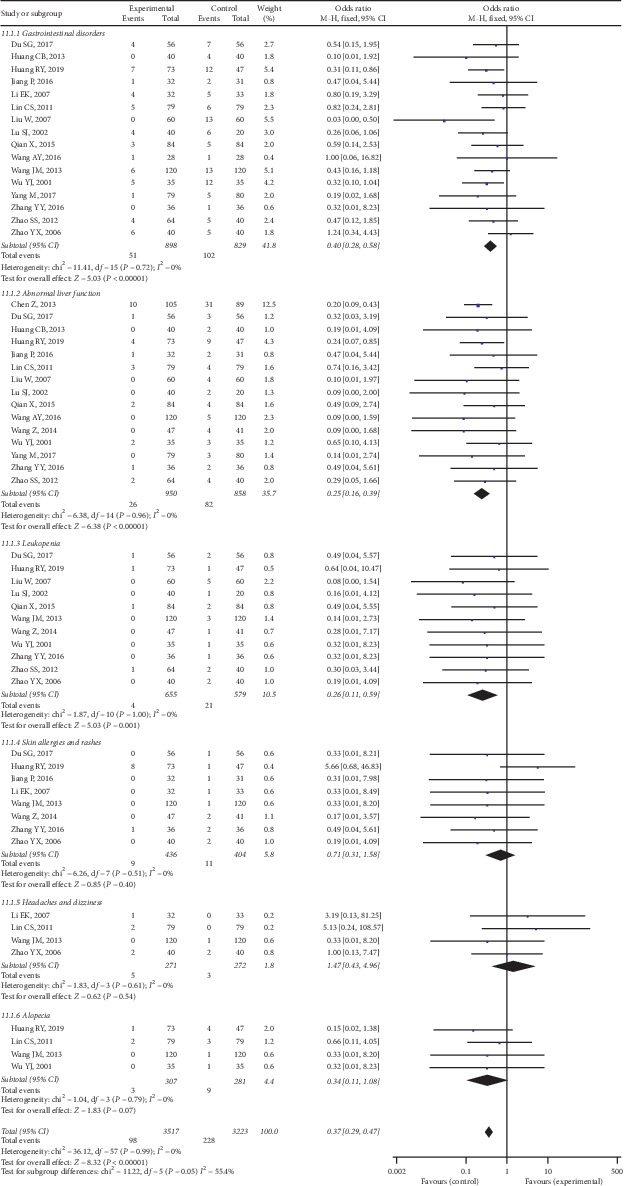
AEs between two groups.

AEs caused by combined TCM-WM or WM alone were reported in 18 of the studies. The most common AEs in both groups were gastrointestinal disorders, abnormal liver function, leukopenia, skin allergies and rashes, headaches and dizziness, and alopecia. Most of the studies were not affected by these AEs; only 6 studies [35, 36, 44, 45, 48–50] reported that some participants withdrew from the trials because of serious AEs. No heterogeneity was identified among the trials (*I*^2^ = 0%, *P* = 0.99) based on a fixed-effect model. As shown in Figure 14, a statistically significant difference was presented between the overall AEs in 2 groups. According to the meta-analysis, the experimental group had fewer AEs than the control group.

### 3.5. Funnel Plot

**Figure 15:**
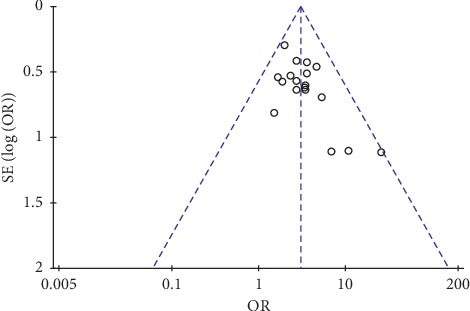
Funnel plot.

TEs were used to measure publication bias. Funnel plot was conducted based on all of studies included (Figure 15). The results revealed that the funnel plot was graphic symmetrical in general and the patterns were concentrated in the middle-upper part except for 3 offsets, which indicated a mild publication bias.

## 4. Discussion

RA is a common internal medical disease mainly affected by both environmental and genetic factors [51]. If not treated promptly, it may lead to joint deformity or even complete loss of joint function, thus affecting the daily activities and working abilities of patients, and have high disability and teratogenic rate [52]. WM treatment plays a role in relieving inflammation, reducing pain, and slowing joint damage; though the overall effects are positive, there are deficiencies, such as more adverse reactions and expensive costs, that ought to by no means be ignored. In recent years, there has been an increase in the use of integrated TCM-WM to treat RA. The integrative medicine combines the advantages of the theoretical experience of TCM with conventional WM, aiming to increase the efficacy, minimize adverse reactions during treatment, and improve prognosis of the diseases.

Early diagnosis and treatment are likely to influence the outcomes of the disease and even the remission conditions [53]. Autoantibodies RF and anti-CCP belong to the seral biomarkers involved in the 2010 ACR/EULAR RA classification criteria, exhibiting essential serodiagnostic utility [54]. Combination of indicators of RF and anti-CCP makes for specific diagnosis of RA [55]. Acute-phase reactants ESR and CRP are important means for assessing the degree of activity of chronic inflammatory lesions as the increases in the levels of these clinical inflammatory markers indicate high disease activity [56]. DAS28 was reported in a mass of daily practice as well as clinical trials in RA [57]. The most common composite index of remission employs the DAS using 44 or 28 joint counts; the latter goes by the name of DAS28, which could monitor the disease evolution. DAS28 < 2.6 is generally considered to be in remission [58]. Therefore, in addition to clinical symptoms and adverse reactions, this meta-analysis was also used to evaluate the effects of integrated TCM-WM on the regulation of these indexes in RA patients, by which providing evidence-based medical basis for the clinical application of integrated medicine. Compared with WM alone, the combination of TCM and WM treatment could increase TEs, and the improvements of TJC, SJC, DMS, GS, DAS28, RF, anti-CCP, ESR, and CRP values were prominent in this study.

Some related findings might provide explanations for the therapeutic effects of integrated TCM-WM treatment in RA. Li et al. [59] pointed out that abnormal cellular immunity, such as high percentages of peripheral blood CD4^+^, CD8^+^, and CD4^+^/CD8^+^ ratio, and increased IgG and IgA levels existed in RA patients. After 1 month of integrated TCM-WM treatment, the CD4^+^/CD8^+^ ratio and the levels of IgG and IgA decreased obviously, demonstrating that combination of TCM and WM could regulate the balance of T-lymphocyte subsets. Other researchers [60] chose RA patients with damp-heat-obstruction symptom pattern as research subjects and divided them into TCM Sanhuangyilong decoction plus MTX group and MTX-only group. It was found that TNF-*α* and IFN-*γ* may play a part in the development of RA. After 4 weeks of treatments, TNF-*α* and IFN-*γ* levels were significantly decreased in Sanhuangyilong decoction plus MTX group, and the differences in TNF-*α* and IFN-*γ* between 2 groups were statistically significant. Moreover, the combined treatment had more clinical benefits than MTX only. Liu et al. [61] compared the treatment characteristics of TCM and WM on the articular cartilage erosion related biochemical and immune factors and found that TCM mainly increased red blood cell count which bounded up with the degree of cartilage damage while platelet count decreased after WM treatment, showing that both TCM and WM could ameliorate cartilage damage in RA, but acted in different ways.

Drug-drug interactions have always been an active area that cannot be ignored in clinical medicine research. Some drugs can be used in combination to obtain an effectiveness that cannot be achieved with TCM or WM alone, but some may cause AEs and even endanger life. As the main means of treating RA, WM may cause a variety of AEs, especially gastrointestinal disorders, abnormal liver function, leukopenia, skin allergies and rashes, headaches and dizziness, and alopecia, which could affect patient compliance to some extent. In contrast, the frequency and severity of AEs in the treatment of integrated medicine were lower than those in WM in this meta-analysis. However, in order to ensure safe medication, we had better continue paying attention to this area. In Taiwan, a multi-TCM/WM interactions database was built to report the prevalence of interactions between TCM and WM, which could issue timely alerts when embedded inside the hospital clinical information system [62].

This study has several strengths: first, since the study included not only Chinese trials but also English trials, we have obtained a greater range of data than any other previous study in China. Moreover, the study did not limit patients to specific TCM or WM treatment options, which means that the results could be applied more extensively to RA patients. Furthermore, we collected as many outcome indicators as possible to acquire a more comprehensive evaluation of the effectiveness of treatment. Still, we are supposed to consider the following limitations: (1) all included studies were conducted in China, so there was a certain racial bias; (2) most of them had no or just a brief description of the principle of randomization, allocation concealment, or blinding; and (3) the TCM or WM regimens involved in the various studies were not entirely consistent, and there were also differences in the dosage and course of treatment under the same regimens, which increased statistical difficulty. Heterogeneities were found in some outcome indicators of this study, which could influence the accuracy and reliability of the results. Correctness of data was first checked to confirm that heterogeneities were not caused by data entry errors. Due to factors such as small sample sizes, loose experimental designs, different treatment durations, and inconsistent interventions, the outcomes were affected to varying degrees, which may also result in certain heterogeneity of results. In order to obtain reliable meta-analysis results, this study used the strategy of changing the statistical effect model. Based on the above, we recommend the following changes in clinical studies: (1) larger sample sizes, multiple centers, and longer follow-up times are required; (2) strict inclusion and exclusion criteria should be developed and outcome assessment and safety analysis need to be standardized; (3) randomization, assignment of concealment, blinding, and other information should be described, and patients who lost follow-up or dropped out of the studies are supposed to be recorded timely, thereby reducing methodological heterogeneity and reporting bias, and further improving the quality of evidence-based medicine research.

After a systematic review of 20 articles with 2269 cases, the study found that comprehensive medical treatment of RA has been widely proved to be therapeutic. Compared with WM, integrated treatment of RA is a more preferable intervening measure, with obvious advantages in improving efficacy and reducing adverse reactions. Nevertheless, prospective, large-sample, and long-term trials are needed in the future.

## 5. Conclusion

This meta-analysis demonstrated the possibility that the combination of TCM and WM for the treatment of RA might be more effective and safer than WM monotherapy. In addition to effectively improving clinical symptoms and reducing laboratory indexes, it may cause fewer side effects. Therefore, we suggest that integrated TCM-WM could be applied to the clinical treatment of RA. Further researches should aim to standardize RA treatment in order to strengthen the basis for combining TCM with WM.

## Figures and Tables

**Table 1:** Study characteristics.

Author	Year	Sample size (male/female)		Age (years)		Disease course		Intervention		Duration	Outcomes
		EG	CG	EG	CG	EG	CG	EG	CG		
Wu et al. [31]	2001	35 (8/27)	35 (7/28)	58.6 ± 2.6	56.7 ± 1.8	42.5 ± 15.1 months	40.0 ± 11.9 months	TWP 10 mg, tid + MTX 7.5 mg, qw + NSAIDs	MTX 15 mg, qw + NSAIDs	3 months	①②③④⑤⑦⑨⑪
Lu et al. [32]	2002	40 (9/31)	20 (5/15)	41.5 ± 11.2	40.6 ± 13.2	2.6 ± 1.2 years	2.8 ± 1.3 years	FS1 30 ml, bid + MTX 5∼10 mg, qw + SSZ 0.5∼1.0 g, tid + NSAIDs	PLB 30 ml, bid + MTX 5∼10 mg, qw + SSZ 0.50∼1.0 g, tid + NSAIDs	24 weeks	①②③④⑤⑦⑨⑩⑪
Zhao and Liu [33]	2006	40 (18/22)	40 (14/26)	31.0 ± 8.9	30.0 ± 9.6	4.0 ± 3.8 years	5.0 ± 4.9 years	TGP 0.6 g, tid + LEF 10 mg, qd	LEF 10 mg, qd	12 weeks	①④⑤⑦⑧⑨⑩⑪
Liu et al. [34]	2007	60 (12/48)	60 (10/50)	44.13 ± 19.29	43.75 ± 14.52	10.5 ± 7.64 years	9.63 ± 7.57 years	QT 5pills, tid + PDN	Voltaren 75 mg, qd + HCQ 0.2 g, qd + MTX 5∼15 mg, im, qw + PDN	20 weeks	①②③⑨⑩⑪
Li et al. [35]	2007	32 (5/27)	33 (4/29)	50 ± 10	50 ± 13	9.3 (4.8, 18.0) years	7.8 (5.5, 11.5) years	CPM 1.5 g, bid + DMARDs or NSAIDs or PDN	PLB 1.5 g, bid + DMARDs or NSAIDs or PDN	24 weeks	①②③⑨⑩⑪
Lin et al. [36]	2011	79 (19/60)	79 (13/66)	51.76 ± 11.67	48.62 ± 13.01	5.46 ± 6.11 years	5.03 ± 4.24 years	KXC 0.3∼0.6 g, tid + MTX 10 mg, qw	MTX 10 mg, qw	12 weeks	①②③④⑤⑥⑦⑧⑨⑪
Zhao and Wang [37]	2012	64 (8/56)	40 (4/36)	42.4 ± 12.6	40.7 ± 11.1	2.2 ± 0.6 years	2.0 ± 0.5 years	CPM 0.6 g, tid + MTX 10 mg, qw	MTX 10 mg, qw	3 months	①⑦⑧⑨⑩⑪
Huang et al. [38]	2013	40	40	—	—	—	—	XC 1.5 g, tid + MTX 10 mg, qw	MTX 10 mg, qw	12 weeks	①②③④⑥⑦⑨⑩⑪
Yu and Yu [39]	2013	120 (38/82)	60 (18/42)	37.1 ± 11.5	36.5 ± 10.4	2.9 ± 1.2 years	2.8 ± 1.2 years	CPM 0.6 g, tid + LEF 20 mg, qd + SSZ 1.0 g, bid + celecoxib 0.2 g, bid	LEF 20 mg, qd + SSZ 1.0 g, bid + celecoxib 0.2 g, bid	3 months	①②③④⑤⑦⑨⑩
Wang et al. [40]	2013	120 (31/89)	120 (33/87)	31.62 ± 14.28	33.93 ± 12.46	6.56 ± 4.63 years	7.17 ± 5.82 years	BQZD 200 ml, bid + MTX 10 mg, qw	MTX 10 mg, qw	24 weeks	①②③④⑤⑥⑨⑩⑪
Chen et al. [41]	2013	105	89	—	—	—	—	TGP 0.6 g, tid + MTX 10 mg, qw + LEF 20 mg, qd	MTX 10 mg, qw + LEF 20 mg, qd	24 weeks	①⑥⑦⑧⑨⑩⑪
Wang et al. [42]	2014	47 (8/39)	41 (6/35)	42.82 ± 12.45	44.78 ± 12.38	3.8 ± 6.2 years	4.0 ± 6.4 years	YTR 0.5 agent, bid + MTX 10 mg, qw + LEF 10 mg, qd	MTX 10 mg, qw + LEF 10 mg, qd	12 weeks	①②③④⑦⑨⑩⑪
Qian et al. [43]	2015	84 (32/52)	84 (33/51)	43	45	0.3–146 months	0.8–142 months	CHD 1agent, qd + MTX 10 mg, qw	MTX 10 mg, qw	1 month	①⑪
Jiang et al. [44]	2016	32 (3/29)	31 (4/27)	41 ± 10	43 ± 10	5.6 ± 1.6 months	5.8 ± 1.9 months	HR 150 ml, bid + MTX 7.5∼12.5 mg, qw + folic acid tablet 10 mg, qw	MTX 7.5∼12.5 mg, qw + folic acid tablet 10 mg, qw	24 weeks	①⑥⑦⑧⑨⑩⑪
Zhang et al. [45]	2016	36	36	42.0 ± 9.6	43.1 ± 9.5	5.6 ± 1.6 months	5.8 ± 1.9 months	HF 150 ml, bid + MTX 7.5∼12.5 mg, qw + folic acid tablet 10 mg, qw	MTX 7.5∼12.5 mg, qw + folic acid tablet 10 mg, qw	24 weeks	①⑦⑧⑨⑩⑪
Wang [46]	2016	28 (10/18)	28 (9/19)	35.5 ± 6.6	35.9 ± 6.9	4.7 ± 2.5 years	4.9 ± 2.8 years	CHD 0.5agent, bid + MTX 10 mg, qw + LEF 10 mg, qd	MTX 10 mg, qw + LEF 10 mg, qd	12 weeks	①⑪
Chen [47]	2016	40 (11/29)	40 (12/28)	37.2 ± 14.6	37.6 ± 11.9	6.6 ± 2.6 years	6.3 ± 3.1 years	CHD + MTX 7.5∼20 mg, qw	MTX 7.5∼20 mg, qw	3 months	①
Du et al. [48]	2017	56 (20/36)	56 (21/35)	33.47 ± 12.37	36.52 ± 14.57	7.12 ± 3.72 years	6.93 ± 4.13 years	CHD 300 ml, bid + MTX 7.5 mg, biw	MTX 7.5 mg, biw	16 weeks	①⑥⑦⑧⑨⑩⑪
Yang et al. [49]	2017	79 (16/63)	80 (18/62)	47.59 ± 14.43	44.70 ± 16.41	6.46 ± 6.92 years	7.18 ± 8.37 years	EAA 30 g, qd + LEF 10 mg, qd + MTX 7.5∼15 mg, qw	LEF 10 mg, qd + MTX 7.5∼15 mg, qw	48 weeks	①②③⑥⑦⑧⑨⑩⑪
Huang et al. [50]	2019	73 (18/55)	47 (7/40)	48.97 ± 10.79	48.53 ± 12.10	32.99 ± 44.21 months	40.56 ± 54.52 months	SIN 120 mg, bid + MTX 10∼15 mg, qw + folic acid tablet 5 mg, bid/tid	LEF 20 mg, qd + MTX 10∼15 mg, qw + folic acid tablet 5 mg, bid/tid	24 weeks	①②③④⑥⑨⑩⑪

Quantitative data are shown as mean ± standard deviation or median (interquartile range) or range. EG: experimental group; CG: control group; TWP: *Tripterygium wilfordii* polyglycoside; MTX: methotrexate; NSAIDs: nonsteroidal anti-inflammatory drugs; FS1: Fengshi no.1; SSZ: sulfasalazine; PLB: placebo; TGP: total glucosides of paeony; LEF: leflunomide; QT: Qingbi Tablet; PDN: prednisone; HCQ: hydroxychloroquine; CPM: Chinese patent medicine; DMARDs: disease-modifying antirheumatic drugs; KXC: Kunxian Capsule; XC: Xinfeng Capsule; BQZD: Bushen Quhan Zhiwang Decoction; YTR: Yangxue Tongluo Recipe; CHD: Chinese herbal decoction; HR: Hebi Recipe; HF: Hebi Formula; EAA: the extract of *Artemisia annua* L.; SIN: sinomenine; ①: therapeutic effects (TEs); ②: tender joint count (TJC); ③: swollen joint count (STC); ④: duration of morning stiffness (DMS); ⑤: grip strength (GS); ⑥: disease activity score in 28 joints (DAS28); ⑦: rheumatoid factor (RF); ⑧: anti-cyclic peptide containing citrulline (anti-CCP); ⑨: erythrocyte sedimentation rate (ESR); ⑩: C-reactive protein (CRP); ⑪: adverse events (AEs).

## Supplementary Materials

Supplementary Materials Supplementary file 1: Search strategies.
