# Supplementary material for: Efficacy and Safety of Integrated Traditional Chinese Medicine and Western Medicine on the Treatment of Rheumatoid Arthritis: A Meta-Analysis
Source: Evid Based Complement Alternat Med. 2020 Apr 2;2020:4348709. doi: 10.1155/2020/4348709 (PMC7154968; doi:10.1155/2020/4348709)
Supplement: Supplementary Materials — Supplementary file 1: Search strategies. [file 4348709.f1.pdf]

Supplementary File 1. Search Strategies

PubMed

(((("Arthritis, Rheumatoid"[Mesh]) OR Rheumatoid Arthritis[Title/Abstract])) AND ((("Medicine, Chinese Traditional"[Mesh]) OR "Drugs, Chinese Herbal"[Mesh]) OR ((((((((((Traditional Chinese Medicine[Title/Abstract]) OR Traditional Medicine, Chinese[Title/Abstract]) OR Chinese Traditional Medicine[Title/Abstract]) OR Chinese Medicine, Traditional[Title/Abstract]) OR Chinese Drugs, Plant[Title/Abstract]) OR Chinese Herbal Drugs[Title/Abstract]) OR Herbal Drugs, Chinese[Title/Abstract]) OR Plant Extracts, Chinese[Title/Abstract]) OR Chinese Plant Extracts[Title/Abstract]) OR Extracts, Chinese Plant[Title/Abstract]) OR Chinese Patent Medicine[Title/Abstract]))) AND ((Randomized controlled trial[Title/Abstract]) OR randomized[Title/Abstract])

EMBASE

#1 ‘rheumatoid arthritis’ / exp

#2 ‘arthritis deformans’ OR ‘arthritis, rheumatoid’ OR ‘arthrosis deformans’ OR ‘beauvais disease’ OR ‘chronic polyarthritis’ OR ‘chronic progressive poly arthritis’ OR ‘chronic progressive polyarthritis’ OR ‘chronic rheumatoid arthritis’ OR ‘disease, beauvais’ OR ‘inflammatory arthritis’ OR ‘polyarthritis, primary chronic’ OR ‘primary chronic polyarthritis’ OR ‘progressive polyarthritis, chronic’ OR rheumarthritis OR ‘rheumatic arthritis’ OR ‘rheumatic polyarthritis’ OR ‘rheumatism, chronic articular’

#3 #1 OR #2

#4 ‘chinese medicine’ / exp

#5 ‘herbaceous agent’ / exp

#6 ‘medicine, chinese traditional’ OR ‘traditional chinese medicine’ OR ‘drugs, chinese herbal’ OR ‘herbaceous drug’ OR ‘herbaceous plant’ OR ‘herbaceous substance’ OR ‘herbal agent’ OR ‘herbal drug’ OR ‘herbal material’ OR ‘herbal medicinal product’ OR ‘herbal preparation’

#7 #4 OR #5 OR #6

#8 ‘randomized controlled trial’ / exp

#9 #3 AND #7 AND #8

the Cochrane Library

#1 MeSH descriptor: [Arthritis, Rheumatoid] explode all trees

#2 (Rheumatoid Arthritis): ti, ab, kw

#3 #1 OR #2

#4 MeSH descriptor: [Medicine, Chinese Traditional] explode all trees

#5 MeSH descriptor: [Drugs, Chinese Herbal] explode all trees

#6 (Traditional Chinese Medicine) OR (Traditional Medicine, Chinese) OR (Chinese Traditional Medicine) OR (Chinese Medicine, Traditional) OR (Chinese Drugs, Plant) OR (Chinese Herbal Drugs) OR (Herbal Drugs, Chinese) OR (Plant Extracts, Chinese) OR (Chinese Plant Extracts) OR (Extracts, Chinese Plant) OR (Chinese Patent Medicine): ti, ab, kw

#7 #4 OR #5 OR #6

#8 #3 AND #7
